# Yellow fever vaccination before and during the covid-19 pandemic in Brazil

**DOI:** 10.11606/s1518-8787.2022056004503

**Published:** 2022-05-18

**Authors:** Tércia Moreira Ribeiro da Silva, Ana Carolina Micheletti Gomide Nogueira de Sá, Elton Junio Sady Prates, Daiana Elias Rodrigues, Thales Philipe Rodrigues da Silva, Fernanda Penido Matozinhos, Ed Wilson Rodrigues Vieira

**Affiliations:** I Universidade Federal de Minas Gerais Escola de Enfermagem Departamento de Enfermagem Materno-Infantil e Saúde Pública Belo Horizonte MG Brasil Universidade Federal de Minas Gerais. Escola de Enfermagem. Departamento de Enfermagem Materno-Infantil e Saúde Pública. Belo Horizonte, MG, Brasil; II Universidade Federal de Minas Gerais Faculdade de Medicina Belo Horizonte MG Brasil Universidade Federal de Minas Gerais. Faculdade de Medicina. Belo Horizonte, MG, Brasil

**Keywords:** Yellow Fever Vaccine, Vaccination Coverage, Immunization Programs, COVID-19, Time Series Studies

## Abstract

**OBJECTIVE:**

To analyze the number of yellow fever vaccine doses administered before and during the covid-19 pandemic in Brazil.

**METHODS:**

This is an ecological, time series study based on data from the National Immunization Program. Differences between the median number of yellow fever vaccine doses administered in Brazil and in its regions before (from April/2019 to March/2020) and after (from April/2020 to March/2021) the implementation of social distancing measures in the country were assessed via the Mann-Whitney test. Prais-Winsten regression models were used for time series analyses.

**RESULTS:**

We found a reduction in the median number of yellow fever vaccine doses administered in Brazil and in its regions: North (-34.71%), Midwest (-21.72%), South (-63.50%), and Southeast (-34.42%) (p < 0.05). Series showed stationary behavior in Brazil and in its five regions during the covid-19 pandemic (p > 0.05). Brazilian states also showed stationary trends, except for two states which recorded an increasing trend in the number of administered yellow fever vaccine doses, namely: Alagoas State (before: β = 64, p = 0.081; after: β = 897, p = 0.039), which became a yellow fever vaccine recommendation zone, and Roraima State (before: β = 68, p = 0.724; after: β = 150, p = 0.000), which intensified yellow fever vaccinations due to a yellow fever case confirmation in a Venezuelan State in 2020.

**CONCLUSION:**

The reduced number of yellow fever vaccine doses administered during the covid-19 pandemic in Brazil may favor the reemergence of urban yellow fever cases in the country.

## INTRODUCTION

National and international health agencies have recommended maintaining the implementation of immunization strategies during the coronavirus disease 2019 (covid-19) pandemic, mainly because immunization is an essential health strategy^1^. However, more than a third of countries in the world have interrupted their immunization services during the covid-19 pandemic^4^. Moreover, fear of getting infected by SARS-CoV-2, which is the etiologic agent of covid-19^5^, has contributed to reducing the demand for vaccination in health services, even in countries which continued to implement immunization strategies.

This process has had a negative impact on the recommended vaccine coverage in several countries and regions worldwide. Consequently, such low vaccination coverage during the covid-19 pandemic may cause yellow fever (YF) outbreaks, according to a study assessing the impact of vaccination interruption during the covid-19 pandemic in 10 countries^6^. In Ethiopia and Nigeria, a one-year delay in campaigns against YF can increase approximately one death per 100,000 people, highlighting the risk of virus circulation in these populations^6^.

Yellow fever is a hemorrhagic disease caused by the yellow fever virus, which stands out among vaccine-preventable infectious diseases^7^. It is endemic to 47 low- and middle-income countries in the African and South American continents. Moreover, YF features varying severity and lethality levels and accounts for at least 60,000 deaths a year^6^. The yellow fever lethality rate in Brazil is also quite high. According to estimates, the disease accounted for 47.8% of death cases in the country from 2000 to 2021, on average^10^.

Yellow fever vaccination was introduced in Brazil by its National Immunization Program (PNI) in 1937. It was provided free of charge by primary care services to the population in the age group between nine months–59 years old. Such a strategy enabled ruling out urban YF in the country and consolidated itself as the main way to control YF^9 , 11^. Based on a systematic immunization schedule, the National Immunization Program ensures the vaccination of individuals who live in or travel to states located in the *Áreas com Recomendação de Vacina* (Vaccine Recommendation Zones – ACRV), in which YF transmission can take place^11 , 12^.

However, PNI has progressively expanded ACRVs in Brazil from 2014 onward. Such an expansion process comprised states and municipalities which, until then, were classified as free from YF virus circulation. It was implemented in response to surveillance strategies aimed at epizootics (death of non-human primates) and the YF epidemic, which started in the Midwest in 2016. The aforementioned epidemic accounted for 2,114 disease cases and for more than 700 deaths, most of which were recorded in regions that, until then, were YF-free. It was the worst YF outbreak in the history of the country^12 , 13^. Since 2020, the National Immunization Program has expanded its yellow fever vaccination recommendation to the entire national territory^11 , 12^.

The increased number of YF cases recorded during this outbreak has raised red flags to the likelihood of urban YF reemergence in Brazil since *Aedes aegypti* - which is a potential vector of the YF virus found in all Brazilian urban regions - can infect individuals with YF who will transmit the virus to other susceptible individuals and, consequently, perpetuate the urban cycle of the YF virus^7 , 10^. Although the immunization of individuals living in, or travelling to, ACRVs in Brazil is mandatory, vaccination coverage rates have remained below the targets established by the Ministry of Health^12^.

Many factors have favored such a vaccination coverage reduction, namely: precariousness of the Brazilian Unified Health System (SUS), implementation of the new immunization information system (SI-PNI), social and cultural aspects affecting vaccination acceptance, and inconstant availability of immunobiological drugs in primary care services^9 , 14^. Furthermore, vaccination coverage in Brazil is heterogeneous^16 , 19^. Thus, investigating and monitoring low vaccination coverage zones is a strategic axis of good management practices focused on immunization programs, as recommended by World Health Organization^20^.

This study aimed to investigate the YF vaccination status in Brazil and time variations in the number of YF vaccine doses administered in Brazilian states and regions before and after the onset of the covid-19 pandemic by considering that the reduction in YF vaccine coverage rates in the country may have been worsened by the covid-19 pandemic and that YF incidence in large-sized cities may favor urban YF reemergence. Assumingly, results in this study may guide health strategies and policies focused on priority geographic zones that have shown decreased rates of YF vaccine doses administered over time.

## METHODS

### Study Design

This is an ecological study conducted with data from the information system of the National Immunization Program (SI-PNI), available at http://sipni.datasus.gov.br/. SI-PNI provides the number of vaccine doses administered countrywide, stratified by month.

### Data Collection

Collected data refer to the number of YF vaccine doses administered to the Brazilian population from April 2019 to March 2021. Data extraction was based on the number of doses administered to the target audience – namely: nine-month- (first dose) and four-year-old children (second dose) – on a monthly basis.

### Variables

The number of administered doses was used as the dependent variable, whereas independent variables comprised geographic information about the five regions in the country (North, Northeast, Midwest, Southeast, and South), all 26 Federation Units and the Federal District, and all 5,568 Brazilian municipalities.

### Statistical Analysis

First, YF vaccine doses administered before (from April 2019 to March 2020) and after (April to March 2021) the implementation of social distancing measures in Brazil and its regions were summed. Next, differences between the median number of doses administered before and after the implementation of social distancing measures were evaluated by the Mann-Whitney U test, by considering interquartile ranges (IQR) at 5% significance level.

Variation rates in the median number of administered doses were estimated by the following equation:

[(median number of doses administered before the implementation of social distancing measures − median number of doses administered after the implementation of social distancing measures)/median number of doses administered before the implementation of social distancing measures x 100].

These analyses were processed in the Statistical Package for Social Sciences software (IBM-SPSS, v.19, IBM, Chicago, IL).

Moreover, time series analysis was used to check the effect of social distancing measures on time series observed for the number of administered YF vaccine doses, based on Prais-Winsten linear regression models^21^.

Time series in the absolute number of YF vaccine doses administered on a monthly basis from April 2019 to March 2020 (before the implementation of social distancing measures) and from April 2020 to March 2021 (during social distancing measures) were estimated. Data analysis also considered the absolute number of doses administered every month countrywide, as well as in its regions (North, Northeast, South, Southeast, and Midwest) and states. Prais-Winsten regression models were used to assess either significant increasing or decreasing trends in the number of administered YF vaccine doses. This model is based on a linear regression analysis. It aims at correcting the autocorrelation effect and is recommended for time series studies^21^.

The indicator of interest (absolute number of administered doses) set for each month was used as the outcome variable in this analysis, whereas the surveyed month was used as an explanatory variable. There was a significant increasing or decreasing trend in the absolute number of doses administered when the model slope was different from zero and its p-value was equal to or lower than 0.05 (p ≤ 0.05). A positive regression coefficient indicates an increased variation in the absolute number of monthly-administered doses within the evaluated period, whereas a negative regression coefficient indicates a reduced variation in this parameter.

A trend in the absolute number of doses administered was considered stationary whenever a statistically insignificant difference in the absolute number of monthly-administered doses was identified (p ≥ 0.05) in the evaluated period. Model accuracy was expressed by the coefficient of determination (R^2^). The Durbin-Watson test was applied to the entire investigated period to check the incidence of autocorrelation in the series^22^.

Time series trend analyses were performed in the professional statistical software Stata, version 14.

### Ethical Aspects

Since this study was based on freely accessible data, it did not require submission to a research ethics committee, as determined by the National Health Council Resolution n. 466/2012.

## RESULTS

In total, 11,499,231 yellow fever vaccine doses were administered countrywide from April 2019 to March 2021, of which 4,533,135 (39.42%) after the implementation of social distancing measures. The median number of YF vaccine doses administered before the implementation of social distancing measures was 518,510 (IQR = 432,140–705,034), whereas the median number of vaccine doses administered when these recommendations were in force was 349,028 (IQR = 306,190–395,746). This outcome has indicated a 48.55% reduction (p = 0.003) in the number of administered YF vaccine doses.

All regions showed a reduced median number of YF vaccine doses administered during social distancing, except for the Northeastern region, which recorded an increased median number of YF vaccine doses administered during this period (p < 0.05).

States in the Northeastern region, such as Alagoas, Ceará, Paraíba, and Pernambuco - which became ACRVs in 2020 - showed an increased median number of YF vaccine doses administered during social distancing. Other Brazilian states showed a reduced median number of administered doses which ranged from 73.93% (in Roraima State) to 2.91% (in Espírito Santo State); p < 0.05 ( Table 1 ).

**Table 1:** Median and rate of variation in the median number of yellow fever vaccine doses administered in the Brazilian population from April 2019 to March 2020 and from April 2020 to March 2021. National Immunization Program, Brazil.

States and Regions	Apr/19–Mar/20 Median (P25–P75)	Apr/20–Mar/21 Median (P25–P75)	Variation (%)	p^a^
Brazil	518,510 (432,140–705,034)	349,028 (306,190–395,746)	-48.55	**0.003**
North	50,108 (44,692–51,908)	32,715 (26,462–36,436)	-34.71	**0.000**
Acre	1,818 (1,645–2,197)	1,080 (975–1,318)	-40.59	0.000
Amapá	1,962 (1,887–7,736)	1,811 (889 – 3,698)	-7.69	0.000
Amazonas	13,900 (12,514–15,589)	10,966 (7,606–12,429)	-21.10	0.000
Pará	16,540 (16,084–17,783)	11,466 (9,239–13,117)	-30.67	0.000
Rondônia	5,486 (4,449–5,835)	3,874 (3,042–4,284)	-29.38	0.000
Roraima	6,623 (5,310–7,217)	1,726 (1,370–2,465)	-73.93	0.000
Tocantins	3,273 (2,629–3,612)	2,815 (2,445–2,958)	-13.99	0.000
Northeast	58,173 (51,450–89,927)	108,070 (91,645–149,778)	+85.77	**0.003**
Alagoas	824 (719–1,414)	5,025 (265 – 8,214)	+509.83	0.000
Bahia	26,101 (22,959–31,814)	17,716 (15,645–19,779)	-32.12	0.000
Ceará	1,519 (1,392–2,253)	16,826 (8,540–31,387)	+1,007.70	0.000
Maranhão	14,954 (13,416–16,586)	9,399 (7,685–12,399)	-37.14	0.000
Paraíba	1,080 (988–1,314)	7,268 (2,601–8,531)	+524.40	0.000
Pernambuco	3,290 (2,833–29,374)	63,087 (31,010–76,894)	+1,817.53	0.000
Piauí	6,381 (5,350–7,382)	5,077 (4,314–5,450)	-20.43	0.000
Rio Grande do Norte	1,596 (1,253–1,674)	1,532 (1,296–2,328)	-4.01	0.000
Sergipe	825 (707–949)	307 (187–334)	-62.78	0.000
Midwest	38,328 (32,905–47,397)	30,002 (26,385–34,161)	-21.72	**0.017**
Distrito Federal	6,876 (6,205–10,984)	6,034 (5,752–7,433)	-12.24	0.000
Goiás	13,850 (12,817–14,678)	10,157 (9,331–12,115)	-26.66	0.000
Mato Grosso	11,186 (9,655–11,945)	8,118 (7,172–8,913)	-27.42	0.000
Mato Grosso do Sul	5,859 (5,292–7,116)	5,117 (4,912–5,754)	-12.66	0.000
Southeast	171,181 (151,379–204,971)	112,257 (98,569–124,014)	-34.42	**0.000**
Espírito Santo	7,981 (7,004–12,891)	7,748 (6,371–8,664)	-2.91	0.000
Minas Gerais	39,856 (33,706–48,029)	28,281 (25,697–32,761)	-29.04	0.000
Rio Janeiro	20,704 (18,955–24,538)	15,645 (12,986–17,591)	-24.43	0.000
São Paulo	105,524 (95,106–123,009)	59,876 (51,960–65,557)	-43.25	0.000
South	174,257 (153,009–272,348)	63,590 (54,659–78,093)	-63.50	**0.000**
Paraná	56,869 (47,954–90,699)	28,450 (22,136–36,574)	-49.97	0.000
Santa Catarina	69,415 (62,682–123,464)	19,441 (16,330–22,289)	-71.99	0.000
Rio Grande do Sul	35,480 (24,488–50,338)	15,609 (13,943–20,944)	-56.00	0.000

P: percentile.

^a^ Mann-Whitney test (difference between medians).

Figure shows the time series referring to the number of YF vaccine doses administered in Brazil and in its regions. There was a reduction in the absolute number of YF vaccine doses administered in April 2020, when the country implemented social distancing measures. Trends in the absolute number of doses administered remained stationary in the two investigated periods in Brazil and in its regions (p ≥ 0.05). However, its slope reduction speed (β) has increased during social distancing.

**Figure:**
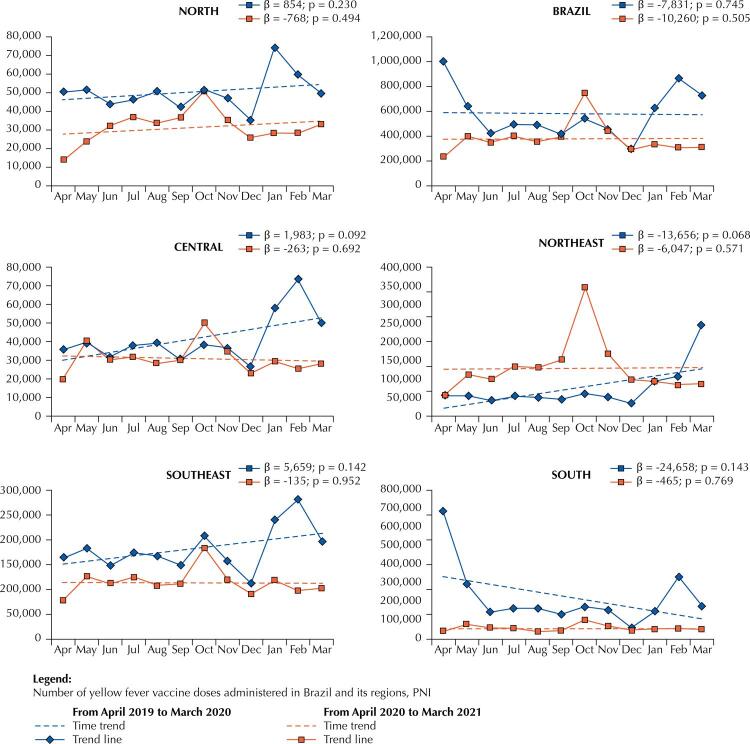
Time trends in the number of yellow fever vaccine doses administered before and after the implementation of social distancing measures in Brazil and in its regions. National Immunization Program (PNI). From April 2019 to March 2021, Brazil.

Tables 2 and 3 show the number of vaccine doses administered per month and the time trends referring to the number of YF vaccine doses administered in Brazilian states during the evaluated periods. The time trend observed in the number of vaccine doses changed from increasing to stationary in the Federal District, as well as in Espírito Santo and Piauí states. Distrito Federal, Espírito Santo, and Rio Grande do Norte showed an increasing trend before the pandemic (p < 0.05) which were stationary after the pandemic (p > 0.05). Alagoas and Roraima had stationary trends before the pandemic (p > 0.05), and increasing ones after it (p < 0.05).

**Table 2:** Time trends in the number of yellow fever vaccine doses administered before the implementation of social distancing measures in Brazil, based on Federation Units. National Immunization Program. From April 2019 to March 2020, Brazil.

States	Apr/19	May/19	Jun/19	Jul/19	Aug/19	Sep/19	Oct/19	Nov/19	Dec/19	Jan/20	Feb/20	Mar/20	p	Slope (β)
Acre	1,790	1,847	1,679	1,917	1,634	1,444	1,550	1,865	1,741	3,065	2,903	2,291	0.118	78
Alagoas	761	777	628	639	705	779	870	993	1,537	1,675	1,698	1,047	0.081	64
Amapá	1,965	2,019	2,092	2,132	2,184	1,289	2,105	1,946	1,616	1,960	1,341	1,018	0.01	-67.87
Amazonas	14,765	14,432	13,045	12,676	15,013	13,369	12,460	11,390	8,263	32,468	19,372	15,782	0.227	580
Bahia	25,532	24,413	16,884	26,068	26,135	22,475	32,732	27,514	15,447	32,161	38,954	30,774	0.066	927
Ceara	2,369	2,589	2,302	2,106	1,524	1,514	1,458	1,382	1,549	1,273	1,219	1,423	0.004	-105
Distrito Federal	6,238	7,136	5,666	6,491	8,656	6,617	8,189	6,194	5,453	11,761	21,773	16,711	0.045	968
Espírito Santo	6,836	7,831	5,883	7,765	8,131	6,844	9,350	10,476	7,486	13,697	25,898	14,291	0.011	1,078
Goiás	13,882	14,693	11,522	13,818	14,635	12,659	13,293	13,533	8,503	21,541	23,329	14,237	0.203	434
Maranhão	17,121	17,877	14,174	15,742	13,496	13,019	15,560	13,390	8,997	14,348	16,659	16,369	0.519	-159
Mato Grosso	10,889	12,017	9,390	11,134	10,452	7,216	11,238	11,319	7,694	15,923	18,555	11,730	0.185	360
Mato Grosso do Sul	5,023	5,846	5,285	6,621	5,969	4,268	5,873	5,758	5,315	8,763	10,194	7,281	0.056	281
Minas Gerais	39,622	46,445	36,274	40,091	35,271	26,432	48,558	33,185	21,038	55,302	63,785	43,780	0.403	888
Pará	16,444	17,421	16,324	17,836	17,789	14,359	16,126	16,070	10,163	19,060	17,766	16,636	0.695	-68
Paraíba	1,295	1,096	974	1,064	1,321	1,356	1,330	882	1,057	1,032	1,111	607	0.116	-33
Paraná	164,056	95,300	63,701	47,877	58,596	48,187	53,704	45,166	26,202	55,142	110,709	76,899	0.253	-5,167
Pernambuco	3,055	2,759	2,491	3,326	3,485	3,291	3,194	2,462	3,289	38,004	40,089	188,155	0.061	12,142
Piauí	5,154	6,134	5,767	6,629	5,837	5,211	7,392	6,893	4,668	7,653	8,866	7,354	0.024	201
Rio de Janeiro	20,843	23,338	18,911	20,566	19,087	15,634	24,938	19,144	13,676	26,616	25,571	21,239	0.512	199
Rio Grande do Norte	1,678	1,650	1,637	1,555	1,829	1,760	1,662	1,216	1,101	1,367	1,433	1,009	0.016	-55
Rio Grande do Sul	28,600	25,282	22,891	60,915	51,414	24,224	40,303	36,537	21,107	34,423	95,760	47,111	0.154	2,449
Rondônia	5,380	5,743	5,121	5,543	4,226	3,567	5,851	5,790	4,056	5,430	6,915	5,854	0.487	60
Roraima	7,100	6,713	3,025	3,794	7,008	6,433	10,568	6,460	7,257	7,589	6,533	4,936	0.724	68
Santa Catarina	495,822	181,492	65,022	67,809	61,903	71,022	86,503	82,199	26,136	67,630	135,785	59,161	0.075	-23,142
São Paulo	96,808	105,584	88,323	105,986	105,465	100,185	124,369	94,539	69,218	145,426	166,707	118,931	0.108	3,505
Sergipe	1,296	1,173	786	957	644	870	865	739	723	702	927	546	0.009	-41
Tocantins	3,055	3,412	2,816	2,567	3,346	1,942	3,355	3,679	2,355	4,842	4,898	3,201	0.081	115

**Table 3:** Time trends in the number of yellow fever vaccine doses administered after the implementation of social distancing measures in Brazil, based on Federation Units. National Immunization Program. From April 2020 to March 2021, Brazil.

States	Apr/20	May/20	Jun/20	Jul/20	Aug/20	Sep/20	Oct/20	Nov/20	Dec/20	Jan/21	Feb/21	Mar/21	p	Slope (β)
Acre	696	953	967	1,071	1,037	1,461	1,896	1,331	1,089	1,281	1,000	1,186	0.212	-0.3
Alagoas	183	201	150	457	3,201	4,512	16,952	8,491	5,538	7,384	6,990	8,843	0.039	897
Amapá	214	192	520	975	817	1,043	1,112	473	869	1,184	1,129	730	0.058	59
Amazonas	1,786	7,913	12,398	12,440	11,863	12,337	20,218	12,451	8,298	6,066	7,504	10,070	0.263	-637
Bahia	13,119	18,899	19,131	21,932	17,963	19,996	24,719	16,629	13,965	16,576	15,335	17,470	0.133	-491
Ceara	1,208	4,436	7,603	11,351	15,863	33,416	83,355	38,839	25,301	17,789	17,861	11,864	0.872	-644
Distrito Federal	5,719	13,080	7,505	6,513	5,934	6,132	11,340	7,219	4,728	5,937	4,415	5,852	0.063	-342
Espirito Santo	4,148	9,094	7,674	8,795	7,663	8,025	13,326	8,274	6,158	7,822	6,305	6,572	0.944	-14
Goiás	6,100	13,851	10,341	11,245	9,974	10,854	16,981	12,405	7,995	9,376	9,316	9,974	0.883	-36
Maranhão	6,039	7,444	10,661	13,533	11,537	12,687	16,073	8,409	6,951	9,195	8,477	9,603	0.374	-369
Mato Grosso	4,997	9,172	7,653	8,345	7,892	8,399	13,653	9,036	6,171	8,546	7,096	7,400	0.532	-126
Mato Grosso do Sul	3,230	5,055	5,264	5,791	4,932	5,117	8,169	6,257	4,375	5,644	4,906	5,117	0.806	-28
Minas Gerais	25,524	38,181	32,384	32,887	28,101	28,462	41,883	28,022	23,577	28,559	23,977	26,216	0.075	-710
Pará	5,796	6,956	10,378	13,458	11,729	13,315	15,493	12,525	8,860	11,203	10,641	12,500	0.888	-53
Paraíba	87	495	1,721	5,241	6,964	6,618	30,159	10,210	7,572	8,605	7,652	8,312	0.232	860
Paraná	21,123	39,622	30,772	29,118	20,423	25,178	43,207	38,509	27,494	28,284	28,616	20,704	0.33	-784
Pernambuco	38,147	76,968	57,166	76,672	69,198	69,009	163,931	77,011	35,487	29,518	25,455	27,963	0.495	-2,694
Piauí	2,410	4,293	5,178	5,465	5,373	5,591	8,624	5,408	4,013	4,977	4,511	4,378	0.429	-137
Rio de Janeiro	7,653	12,798	12,094	15,373	15,917	16,124	34,457	19,329	13,553	17,760	14,551	17,086	0.298	642
Rio Grande do Norte	140	263	1,254	1,443	1,851	2,131	4,446	2,612	2,394	1,424	1,491	1,573	0.884	-28
Rio Grande do Sul	12,745	21,885	18,121	17,521	13,776	15,615	36,966	23,411	14,121	15,603	13,884	14,739	0.51	-520
Rondônia	2,556	4,100	3,871	4,364	3,981	3,877	5,714	4,346	3,015	2,805	3,124	3,364	0.145	-145
Roraima	751	1,101	1,340	1,736	1,677	1,880	2,430	1,462	1,717	2,896	2,584	2,477	0	150
Santa Catarina	19,798	27,866	20,710	19,085	13,339	15,606	29,530	19,003	12,464	18,503	22,290	22,287	0.762	-140
São Paulo	40,687	66,011	60,152	68,357	56,159	59,600	94,071	64,196	48,003	63,606	52,734	51,703	0.939	-88
Sergipe	88	108	170	241	339	300	315	457	315	342	320	251	0.979	-0,5
Tocantins	2,141	3,006	2,851	2,939	2,525	2,965	4,208	2,523	1,995	2,887	2,419	2,779	0.874	-8

## DISCUSSION

There was a significant decrease in the median number of YF vaccine doses administered in Brazil and its Northern, Midwestern, Southern, and Southeastern regions, as well as an increase in the median number of YF vaccine doses administered in the Northeast after the adoption of non-pharmacological measures in response to the covid-19 pandemic. The time trends observed for the number of YF vaccine doses administered in Brazil and its Southeastern, Southern, and Midwestern regions were stationary. However, they showed a faster slope reduction speed during the implementation of social distancing measures imposed by the covid-19 pandemic.

Alagoas State became an ACRV in 2020^10^ and Roraima State has intensified yellow fever vaccine administrations due to a YF case confirmed in the Venezuelan State of Bolívar (bordering Roraima State) in 2020^7^, which may explain the increasing trend in the number of vaccine doses administered in these Brazilian states. Furthermore, these findings can be explained by the sharp decrease in the number of YF vaccine doses administered in April 2020, which preceded the abrupt increase in the number of YF vaccine doses administered in the subsequent months until the end of the evaluated period.

Findings in this study have indicated that some states in ACRVs, and those which reported recent cases of wild YF and epizootics, showed decline trends in the number of YF vaccine doses administered within the analyzed period. Santa Catarina State, in Southern Brazil, reported 151 epizootic cases and 26 YF cases in humans from July 2020 to January 2021, indicating YF virus circulation in the State^10 , 23^. Therefore, the decreasing trend in the number of YF vaccine doses identified in this study points toward the risk of sustained YF virus transmission in Santa Catarina State, as well as a risk of urban YF incidence since approximately 55% of its population lives in urban areas^24^.

Moreover, Santa Catarina, Paraná, and Rio Grande do Sul States, which are also located in Southern Brazil, became ACRVs in 2017, when evidence of YF virus circulation was found and its likely dispersion route, identified: it started in areas of typical Atlantic Forest vegetation in São Paulo State (Southeastern Brazil), reaching the South^11^.

Accordingly, the evidence of YF virus circulation associated with a reduction in the median number of administered YF vaccine doses and the downward trend in the number of doses administered in the population living in the South has evidenced the risk of sustained YF virus transmission, which can lead to increasing YF-related mortality rates in humans. The inclusion of Alagoas, Ceará, Maranhão, Paraíba, and Pernambuco States (Northeastern Brazil) in the ACRV^11^ group in 2019 may have contributed to the increasing trend in the number of YF vaccine doses administered throughout the sanitary measures adopted during the covid-19 pandemic.

However, in addition to studies focused on investigating the administration of YF vaccine doses, it is necessary to conduct research focused on assessing YF vaccine coverage to assess whether the target population living in these states was properly immunized and reached the vaccination coverage goal of at least 95% set by the PNI^11 , 25^.

Although YF cases in humans have not yet been identified in these Northeastern states, they have become ACRVs, as determined by the Secretariat of Health Surveillance^11^. This strategy was established in response to the progressive increase of YF and epizootic cases in states outside the Amazon region, which, until then, was the only region within the Brazilian territory known to be endemic for YF^7 , 12^. In addition to the progressive increase in the number of YF cases, an outbreak took place in Brazil between 2016 and 2018. It accounted for the largest number of reported cases in the history of the country and favored YF virus circulation in areas which, until then, were free from its circulation^13^. There were also records of YF cases and death of foreign individuals who were in Brazil at the time of the outbreak^12^.

Although the covid-19 pandemic also plagued the richest regions in Brazil, it is worth emphasizing that it has worsened health inequalities and increased social and ethnic-racial disparities in the country. It mainly affected its poorest regions, such as Northern Brazil^26^. It historically shows the worst immunization indicators in the country, in addition to precarious conditions of primary care services, which account for providing immunobiological drugs to the population for free^16 , 18^. Associated with these factors, the collapse of health services in some Northern states, due to the high demand for hospital beds for patients with covid-19, may have contributed to reducing the population’s demand for immunization services^27 , 28^ and directly impacting the number of YF vaccine doses administered in the region.

Despite increasing trends in the number of YF vaccine doses administered in the Northern region, it is worth emphasizing that we observed fluctuations. April 2020 recorded an abrupt drop in the number of administered YF vaccine doses, though there was a progressive increase in the number of YF vaccine doses administered after April. However, it was insufficient to reach the number of doses administered before the pandemic, which resulted in a 54.22% decrease in the median number of administered YF vaccine doses.

More than a year after the first case of covid-19 in Brazil, immunization rates against this disease are still low in the Brazilian population^29^. Moreover, high transmission and sustained mortality estimates point toward the long-term maintenance of social distancing strategies^30^. Given this scenario, it is necessary to adopt health strategies and policies capable of ensuring the population’s access to YF vaccination. Otherwise, the country will be at risk of living with overlapping epidemics (covid-19 and YF cases and deaths) which may worsen the severe health crisis observed in the country. Furthermore, it is essential to identify the states and regions showing a decreasing trend in the number of administered YF vaccine doses to help to develop strategies focused on improving YF-immunization indicators and reduce the likelihood of YF cases in humans and urban YF reemergence.

Among the limitations of this study, it is worth mentioning its information bias, which is intrinsic to studies conducted with secondary data. However, we used population data available for the investigated period and the generalization of results was relatively safe for national estimates. Moreover, we employed methodological rigor at all stages of this study to control for biases. Also, it is worth emphasizing that, to the best of our knowledge, this study was the first to portray the yellow fever vaccine status in Brazil before and after the covid-19 pandemic.

Assumingly, results in this study may contribute to surveillance strategies focused on YF and epizootic cases to point out priority areas for the establishment of health strategies and policies focused on this particular issue. Furthermore, this study stood out for analyzing variations in YF vaccine dose indicators, both based on location and time, and it enabled us to identify health inequalities and YF vaccination rates before and during the pandemic.

Significant inequalities mark Brazil. Thus, given its continental territorial extension, it is necessary to identify the areas mostly affected by decreased vaccination coverage during the covid-19 pandemic. Therefore, this research can contribute to identifying priority areas that are mostly vulnerable to low YF vaccine coverage. It is worth emphasizing that immunization is essential to achieving health equity. Therefore, identifying vulnerable areas can help to guide public policies and health strategies to improve immunization indicators, which is a goal included in the 2030 United Nations’ Agenda for Sustainable Development Goals.

We found evidence of worsened YF vaccine indicators in Brazil after the adoption of the sanitary measures set to cope with the covid-19 pandemic. Due to the worrying scenario of expansion in YF virus circulation areas in Brazil, as well as the reduced number of YF vaccine doses administered during the covid-19 pandemic, it is necessary to adopt health strategies and policies focused on improving these immunization indicators, especially in states and regions in which YF or epizootic cases were identified. Furthermore, it is imperative to identify areas showing the worst immunization indicators to support surveillance actions and public policies, as well as to reduce inequalities.
